# Language and Intelligence: A Relationship Supporting the Embodied Cognition Hypothesis

**DOI:** 10.3390/jintelligence10030042

**Published:** 2022-07-14

**Authors:** Attà Negri, Marco Castiglioni, Cristina Liviana Caldiroli, Arianna Barazzetti

**Affiliations:** 1 Department of Human and Social Sciences, University of Bergamo, 24129 Bergamo, Italy; arianna.barazzetti@unibg.it; 2 Department of Human Sciences for Education, University of Milano-Bicocca, 20126 Milan, Italy; marco.castiglioni@unimib.it (M.C.); cristina.caldiroli@unimib.it (C.L.C.)

**Keywords:** embodied cognition, intelligence, enaction, linguistic analysis, referential process

## Abstract

Cognitive science has gathered robust evidence supporting the hypothesis that cognitive processes do not occur in an amodal format but take shape through the activation of the sensorimotor systems of the agent body, which works as simulation system upon which concepts, words, and thought are based. However, studies that have investigated the relationship between language and cognitive processes, as both embedded processes, are very rare. In this study, we investigated the hypothesis that intelligence is associated with referential competence, conceived as the ability to find words to refer to our subjective and perceptual experience, and to evoke understanding of this experience in the listener. We administered the WAIS-IV test to 32 nonclinical subjects and collected autobiographical narratives from them through the Relationship Anecdotes Paradigm Interview. The narratives were analyzed linguistically by applying computerized measures of referential competence. Intelligence scores were found to correlate with the use in narratives of words related to somatic and sensory sensations, while they were not associated with other measures of referential competence related to more abstract domains of experience or based on vivid or reflective dimensions of language style. The results support the hypothesis that sensorimotor schemas have an intrinsic role in language and cognition.

## 1. Introduction

In recent decades, a growing number of neuroscientists, cognitive scientists, psychologists, linguists, and philosophers of mind have supported the embodied cognition hypothesis. The “embodiment” perspective rejects the metaphors used by classical cognitive science to represent the mind: the sandwich and computer models. The first model views perception, cognition, and action as three events and processes that follow one another temporally in a linear fashion without the possibility of feedback; in other words, the sandwich model views perception and action as separate processes located at opposite poles and representing the input and output of the cognitive system (Gallistel 1980; Hurley 2001; MacKay 1987); the body is a mere tool of detection and execution in the service of cognition. In the computer model, the mind is seen as a computer-like computational system that transforms inputs into abstract, amodal symbols representing the external world and performs various computations on them to produce outputs; the computer’s operations are taken to be the underlying operations of human cognition (Pfeifer and Scheier 2001); the body is simply the receiver of input information, the brain is the processor hardware, and the mental representations with their rules of connection and computation are the software; the activity of the mind, thus, is different and disconnected from both the body and the external world, and cognitive processes are inaccessible to personal awareness and consciousness (Thompson 2007). Furthermore, based on the multiple realizability assumption (Bickle 2020), due to the abstract nature of cognition, it is irrelevant which kind of physical support realizes a certain cognitive function: it might be a human brain, a computer, a robot, or anything else.

According to several authors, these models present many limitations. For example, the serialized process of the sandwich model (perception-cognition-action) would not be dynamic enough to cope with the urgency of taking an action in the complex scenarios of everyday life. In the time it takes to build a representation and plan an action, integrating the necessary information, the context might have changed (Burr 2017; Hurley 1998). Moreover, the growing body of neurophysiological data is difficult to reconcile with the assumption that sensorimotor processes are separate from other processing steps and localized in well-demarcated regions (Cisek 2007; Cisek and Kalaska 2010; Levy and Glimcher 2012; Padoa-Schioppa 2011). On the other hand, the computer model does not consider the crucial importance of the hardware of cognition. The scientific evidence is incompatible with the idea that cognition takes shape almost independently of both bodily/sensory processes and the surrounding environment. Humans are not observers who engage in understanding the environment and others from a disembodied, third-person position (Thompson 2007).

In the early 1990s, several researchers proposed an alternative theory: the embodied cognition model which asserts the close relationship between acting and thinking. Cognition is a form of embodied action. *Embodied* means that not only the brain but also the whole body is important. Action because the ability to act in the world—agency—is central. Cognition is an expression of our bodily agency. We inhabit a meaningful world because we enact meaning. To be human, the individual needs shared bodily practices and to inhabit the world of culture. For the embodied cognition model, the brain is a necessary condition for mind and meaning. Enculturation is a necessary enabling condition for the brain (Thompson 2007). Higher cognitive processes (i.e., how we think, make decisions, or simply live in a society) are highly dependent and based on sensory and motor processes as well as how the agent interacts with the environment around them. In other words, between perceptual, cognitive, and motor processes, there is no hierarchical relationship or temporal sequence, as in the sandwich model, but rather circularity and overlapping such that action influences both perception and abstract thought and vice versa (e.g., Nöe 2004). Moreover, rejecting the computer metaphor, embodied cognition replaces the concept of amodal computation with that of embodied simulation: cognition does not take shape thanks to the processing of abstract and amodal propositions, but through the reactivation of sensorimotor schemes that are exploited to make sense of what happens in fields and levels of experience different from those in which these schemes have been developed (e.g., Barsalou 1999; Borghi and Binkofski 2014; Brooks 1991).

### 1.1. Relationship between Mind, Body, and Environment

Contrasting the two main classical representational view of cognition, embodied cognition approach is based on sensorimotor coupling of living beings and environment. Organisms always perceive the objects as opportunities for interaction with their body. They do not perceive the realty in passive manner, but through their ongoing bodily activity (Galbusera and Fuchs 2013). Embodied cognition points out that there is no mind separate from the mind–body–environment system. According to Gregory Bateson, mind includes “the pathways of all unconscious mentation—both automatic and repressed, neural and hormonal. Mind is not bounded by skin but includes all external pathways along which information can travel” (Bateson 1972, p. 325). To describe this connection, Bateson used the iconic example of a blind man using his stick to take in information about the environment (Bateson 1972, p. 324): where does his self begins and ends in the process of understanding the world? Where are the boundaries between man’s body and his brain? And between the stick and these surrounding? Or is mind present in the entire interaction between his body, stick and environment?

In Bateson’s perspective, not only our bodies, but also our subjective experience of the world plays a fundamental role in defining and constituting the dynamics of cognition, action, and perception. According to Thompson, subjective experience, and embodiment are interconnected aspects, both central to cognition (2007). Varela et al. (1991) called this approach enactivism. The enactive approach views humans as self-organizing dynamic systems, capable of preserving their identity by adjusting their behavior in relation to perturbations in the environment. This property is called adaptability or the ability of an organism to regulate itself with respect to the boundaries of its viability (Di Paolo 2005). Thus, the world meaning is constituted by the recursive and dynamic interaction between adaptive systems and environment. From this perspective, cognitive processes emerge from recurrent sensorimotor interactions involving the environment, the body, and the brain (Clark 1998, 2008; Fuchs 2011; Fuchs and De Jaegher 2009); movements and action play a central role in high-function processes of human beings and in development of meaning in experience (Nöe 2004). In general, the enactive approach emphasizes the person’s point of view as an autonomous agent who interacts with the world in terms of affordances (Chemero 2009). In this sense, the mind is enactive and for this is intrinsically embodied.

### 1.2. Embodiement of Concepts, Language, and Self

Many lines of research are guided by embodied cognition, enaction, and adaptability. Of particular interest are the studies that address one of the most complex and sophisticated capacities of humans, the ability to form and use concepts. Concepts are the basic units of our knowledge and the building blocks of our thinking. The use of concepts is the basis of intelligent behavior. We need concepts to organize our surroundings, to know how to react to others, how to use objects, how to orient ourselves in space and time.

According to George Herbert Mead (1934), concepts are not representations of objects, but something more akin to the ability to interact with them and thus aimed at action. For example, the concept of “dog” is not a representation of the animal, but rather corresponds to an intricate set of practical knowledge about the dog, including ways of interacting with it. According to the embodied and grounded approach, concepts can be defined as the reactivation of the neural activation pattern that occurs when objects and entities from the external world are experienced. This view is supported by numerous empirical studies. For example, using brain imaging techniques, many researchers have shown that during the processing of linguistic material that includes concepts related to various actions, the effector areas of premotor and motor areas are activated to some extent (e.g., Gallese and Lakoff 2005; Glenberg 2007; Hauk et al. 2004; Tettamanti et al. 2005). In other words, if seeing a couch activates the perceptual system, prepares us for the action of sitting and anticipates a mental state of rest. Thus, our concept of a couch, and the word “couch,” reactivate the same systems to adequately prepare us for interaction with any couch we might be dealing with. In this view, concepts are multimodal and not amodal or unimodal. The information contained in concepts corresponds to the activation of a distributed network of different modalities: tactile, olfactory, visual, etc.; thus, concepts are not abstract, but perceptual symbols (e.g., Barsalou 1999; Lakoff and Johnson 1980).

The embodied nature of concepts and language is based on the hypothesis of simulation through our bodies (Barsalou 1999; Lakoff and Johnson 1980; Rizzolatti et al. 1996). More specifically, Glenberg and Gallese (2012) proposed the action-based language theory that has been supported by a large amount of empirical evidence. In this theory concepts are conceived as possible models of action so that when we understand language, we make predictions about the effects that may follow from our action. In this sense, there is a strong link between concepts, words, and action: when we use concepts, we recall the sensorimotor experience of specific objects or events to which they refer, and it is always through the embodied simulation that we understand the meaning of the words; when we read the word “walk”, we activate the neural circuit related to feet and legs because we simulate the action. In this sense, the simulation enabled by words and concepts helps us to think more than vice versa (e.g., Vygotsky 1962) or using Bucci and Freedman’s words (1978), “we do not know what we are talking about until after we have put it into words”.

García and Ibáñez (2018) report extensive evidence to support the hypothesis of a close relationship between movement skills and language. In their systematic review, they found that “damage to critical hubs, such as motor cortex and basal ganglia, can distinctly impair functionally germane linguistic subdomains” (p. 1). In other words, movement disorders normally lead to specific language deficits.

While sensorimotor encoding is particularly evident and proven in the case of concrete words and concepts, for several authors (Barsalou and Wiemer-Hastings 2005; Caruana and Borghi 2016; Chartrand and Bargh 1999; Gallese 2008; Lakoff and Johnson 1980), it is also central for the abstract concepts and words, in which sensorimotor encoding provides metaphorical material used by decontextualized thinking; in addition, abstract words and concepts are based on multimodal representations that combine encodings of sensorimotor experiences with those derived from other domains of experience such as emotional, social, and linguistic.

Finally, following the embodied theories also our narrations are therefore also structured by the sequence of experiences embodied inasmuch our perceptions and actions are all precedent to the narrative sense of the Self (Menary 2008). Mark Slors has shown that “individual sense perceptions acquire their full sense only as part of a sequence of perceptions portraying a body’s movements through space, individual feelings acquire their full sense only in connection with what evoked them and what they produce” (Slors 1998). The self-narrative stems from a sequence of perceptive, bodily, and action experiences.

### 1.3. Embodied Intelligence

In scientific literature, there have been several attempts to provide a definition of intelligence. Sternberg (2000) published an extensive review of these attempts, showing that some of the definitions given are complementary and some mutually exclusive. The lack of unanimous consensus around the definition of intelligence stems from the unobservable nature of intelligence, which can only be inferred from behaviors. This is also the reason why many assessment theories and techniques have developed over time. Within this plurality and multiplicity of theoretical models, we can distinguish two polarities: on the one hand, theories that consider intelligence primarily as a global, unitary, hierarchically superordinate, abstract, rational, and amodal capacity; on the other hand, theories that conceive intelligence as a set of multiple specific and modular abilities that tend to be modality-specific, practical, concrete, embodied, and grounded.

In most of the past century prevailed the first polarity where the intelligence was operationalized as global and abstract factor (e.g., Spearman 1927; Sternberg 1987; Thurstone 1938). For example, Wechsler (1944, p. 3) defined intelligence as a “global capacity of a person to act purposefully, to think rationally, and to deal effectively with his environment”, Bayley (1970, p. 1171) as “the processes through which the human organism develops from concrete to increasingly abstract mental functions” and Terman (1925, p. 254) as “the ability to engage in abstract thinking”. Similarly, Bruner’s (1966) symbolic representational mode and Piaget’s (1950) formal operational stage—the highest levels of intellectual functioning—involved the ability to deal with logical relations at the symbolic level, regardless of the specific concrete content.

Over the past fifty years, the study of intelligence has shifted primarily to the idea of intelligence as based on a distributed, grounded, and embodied system. Some authors (e.g., Brooks 1991; Guilford 1967; Schneider and McGrew 2012; Sternberg 1996) maintained that it is necessary to investigate relatively independent and specific cognitive abilities that combined may contribute to the general intelligence needed to deal with problems in the environment. For example, Sternberg (2003) thought that the easiest way to measure intelligence was to analyze the degree of adaptation of people into their social context, instead of using tests. He says, “I prefer to refer to it as ‘successful intelligence.’ And the reason is that the emphasis is on the use of your intelligence to achieve success in your life. So, I define it as your skill in achieving whatever it is you want to attain in your life within your sociocultural context”. This implies that intelligence is not a constant component, but that it is dynamic and constantly adapting, and this adaptation depends on the reality and importance that people give it, as well as their motivations and intentionality.

More specifically, in the embodied cognition approach intelligence cannot develop without an embodiment or interaction with the environment. Through embodiment, intelligent agents carry out actions and affect the environment. The response of the environment is registered through bodily sensors. At the same time, the body is a part of the environment that can be perceived, shaped, and learned by intelligence. Intelligent behavior is always considered the outcome of the coupling between the constraints of the agent’s body (the perceptual and motor system, and their brain) and the environment. Thus, movement and bodily interactions constitute the basic elements of sense-making processes with multiple and circular feedbacks between the perceptual, emotional, and cognitive levels (Koch et al. 2007; Niedenthal et al. 2005; Ritter and Graff Low 1996).

Unfortunately, the intelligence tests that have been developed over time have only been partially updated to measure this new embodied understanding of intelligent behavior. In particular, the fourth edition of the Wechsler Adult Intelligence Scale (WAIS-IV; Wechsler 2008) has only partially addressed this gap by introducing and modifying some subtests to expand the number of modality-specific cognitive skills measured that in previous versions were not fully measured, and calculating four partial sub-indices (Verbal Comprehension, Perceptual Reasoning, Working Memory, Processing Speed) considered more reliable measures than IQ, especially in profiles with large discrepancies. However, we consider that Bucci and Freedman’s (1978) evaluation of this test is also applicable to the current version of the scale: The authors believe the WAIS focuses mainly on abstract verbal ability (like in the Vocabulary and Information subtests), associations between words (as in the Similarities subtest), abstract visuo-perceptive reasoning (as in Block Design, Matrix Reasoning, Visual Puzzles, Figure Weights, Picture Completion), decontextualized memory and computation processes (Digit Span, Arithmetic, Letter–Number Sequencing), and recognition of simple and abstract visual patterns (like in Symbol Search, Coding, and Cancellation); in other words, the cognitive skills measured are mainly abstract, decontextualized, and in many cases, far from the sensorimotor coding on which the cognitive skills are based according to the embodied approach. Only the Comprehension subtests requires that responders address themselves to specific situations which they may not have confronted before, but even in this case, the focus is the rational reasoning ability and not the situated and embodied people’s problem solving. It is based on these characteristics that some studies have found some partial limitations in this test in terms of predictive, ecological, and external validity with respect to real everyday cognitive abilities (Groth-Marnat and Baker 2003; Loring and Bauer 2010; Watt et al. 2016).

### 1.4. Referential Competence and Embodied Intelligence

One of the innovative ways to investigate the embodiment of human mentation is proposed by Bucci and colleagues (Bucci 1984, 1997, 2021a, 2021b, 2021c; Bucci et al. 2016; Christian et al. 2021; Mariani et al. 2013; Negri and Ongis 2021; Negri et al. 2019, 2020) and consists of analyzing the content and stylistic characteristics of spoken language. Indeed, the words we choose when we speak and the way we use them reflect both the degree of internal connection between the abstract symbolic levels and the more concrete, embodied levels of our subjective experience, and the degree of interpersonal connection with others, since the quality of the words we use allows us to convey and make others live our experience in a more or less effective way. Bucci and Freedman (1978) refer to this quality of language as referential competence defined as the “degree of integration of symbolic and iconic representation systems, i.e., the strength of the symbol-referent links in semantic memory” (p. 594) or in other words, “the ability to find words for objects, feelings, relationships, etc., i.e., the strength of the word-referent link” (p. 595). People vary greatly in their ability to find the right words to express their meaning, words which will evoke understanding of this meaning in the listener. The commonly used verbal intelligence tests do not tap this ability to use words to refer to object, while they provide measure for the abstract verbal competence related to the ability to handle semantic and syntactic relations between verbal symbols, the degree of abstractness of the verbal systems, and the efficiency of retrieval processes.

The referential competence is a measure of the embodiment of the mentation in general, and specifically of the intelligence. Indeed, the words used are the more effective the more they are connected to bodily, sensory, and motor processes related to the experience we have of the objects to which they refer. The more our speech is specific, clear, concrete, and evocative of visual and sensory images, the more we will be able to convey to the listener the experience we are having. Bucci (1984, 1997, 2021a, 2021b, 2021c) investigating the relationship between mentation and language developed the multiple code theory of human processing and called “referential process” the process of connecting nonverbal experiences, words, and objects. The referential competence is the measure of the outcome of this process and, thus, can be considered an index of embedded intelligence, in that it allows us to deal adaptively and efficiently with problems posed by the physical and social environment in which we are embedded.

In 1978, Bucci and Freedman conducted a study to investigate the relationship between referential competence, language style, intelligence, and hand movements. The referential competence measured using deviation of color naming from word-reading times on the Stroop Color-Word test was shown to be unrelated to verbal abstract intelligence as measured by Vocabulary, Comprehension and Similarity subtests of the 1995 version of WAIS and to the verbal fluency as measured at Stroop test in term of mean word-reading times. Instead, participants with high verbal competence during their five-minute monologue about interesting or dramatic personal experiences produced approximately three times more object-focused hand movements and their narration was specific, vivid, and objective in contrast to the subjective and general verbal material in the low referential group. The lack of association between intelligence and referential competence was explained by the authors by highlighting the abstract verbal nature of the cognitive abilities measured by the WAIS test.

### 1.5. Purposes of the Study

Since Bucci’s pioneering studies (Bucci and Freedman 1978; Bucci 1984), no further research has been conducted on the relationship between intelligence and referential competence. In the meantime, new more sophisticated measures of both constructs have been developed.

The WAIS is now in its fourth edition (Wechsler 2008) and has been modified, albeit only partially, to measure more specifically some of the major situated and perceptually based abilities that shape intelligent behavior. For example, by adopting the CHC model of intelligence (McGrew 2005), the WAIS-IV measures five broad abilities (visual processing, short-term memory, processing speed, crystallized intelligence, and fluid intelligence) and ten narrow abilities (Visualization, Closure Speed, Working Memory, Memory Span, Perceptual Speed, Test Pacing, Lexical Knowledge, General Information, Induction, Quantitative Reasoning). Despite these changes, many situated, and modality-specific skills are not measured (for example, Auditory processing; Domain-Specific Knowledge; Reaction and Decision Speed; Psychomotor Speed; Kinesthetic, Olfactory, Tactile, Psychomotor Abilities) and the current subtests, especially verbal ones, measure abstract and rational rather than situated and embedded skills.

With respect to referential competence, over the past forty years the international research group on referential process, led by Wilma Bucci, has developed several manual, self-report, and computerized instruments to measure this construct in various language (Bucci 2021b; Mariani et al. 2013; Maskit 2021; Maskit and Murphy 2011; Maskit et al. 2012; Negri et al. 2019, 2020; see also the method section). In particular, several computerized linguistic measures have been developed to detect the referential competence in various domains and by various linguistic aspects. The dictionary of the referential activity (WRAD) and the Reflection and Reorganization Function (WRRL) measure the referential competence more in terms of the linguistic style, that is, how the speaker uses words to convey a certain meaning; the dictionaries of affects (AFFD), refection words (RefD), bodily and somatic sensations (SenSD) measure referential competence more in term of verbal content, that is, the type of words most used by the speaker (see the method section for the operational definition).

The aim of the present work was to replicate Bucci and Freedman’s (1978) study using the updated tools for measuring intelligence (WAIS-IV) and referential competence (the computerized linguistic measures of referential process). Based on many studies on embodied cognition that provided robust empirical evidence about the fact that cognition development is based on sensory and motor interactions with the world (Barsalou 1999; Borghi and Binkofski 2014), we hypothesized that subjects with greater referential competence would show higher levels of intelligence. To test the hypothesis, we administered the WAIS-IV (Wechsler 2008) to obtain a measure of participants’ intelligence, and the Relationship Anecdotes Paradigm (RAP) interview (Luborsky and Crits-Christoph 1990) to collect autobiographical narratives about relational episodes that participants considered significant and interesting to tell about themselves.

Our prediction was that the sensorimotor self (Clark 2008; Glenberg and Gallese 2012; Yu and Smith 2012) emerging in self-narratives and observed in language style would correlate with Intelligence Quotient and its sub-indexes more related to performance and perception in space. More specifically, we expected that referential competence about sensory and somatic states and sensations, as measured by calculating the proportion of sensory and somatic words (SenSD) in self-narratives, would correlate with IQ, or at least with PRI, WMI, PSI, the indices most related to performance and perception in space. As for the other indices of referential competence, both content indices (dictionaries of affect and reflection: AffD and RefD) and language style (dictionaries of referential activity and reflection/reorganization function: WRAD and WRRL) indices, we expected that they would be neither positively, nor negatively associated with intelligence indices. In fact, as Bucci and Freedman (1978) found, referential competence that passes through language style, i.e., through the situated, concrete, and contextualized use of words (WRAD), should not be correlated with abstract, decontextualized cognitive competence–be it verbal, perceptual, mental, or performance—as measured by the WAIS. Similarly, we expected that words related to abstract reflection (as measured by the Dictionary of Reflection, RefD), emotional sensations (as measured by the Dictionary of Affect, AffD), and reorganization and reflection style (as measured by the Dictionary of Reflection and Reorganization, WRRL) would not be associated with intelligence indices because they do not directly express sensorimotor sensations.

## 2. Materials and Methods

### 2.1. Procedures and Participants

After reading a brief presentation of the study, the potential participants were invited to a quiet room of the university campus where they expressed their written informed consent to participate in the study. The exclusion criteria were two: (a) being an undergraduate student in psychology or being a psychologist, (b) having a score above the clinical cut offs on the Symptom Check List-90-R (SCL-90_R; Derogatis 1992), which was the first self-report test that was required to be completed.

In total, 32 Italian nonclinical participants (17 females) were recruited via community outreach (e.g., referrals, snowball sampling); their age ranged from 21 to 67 years (*M* = 33.8, *SD* = 14.32), their education was middle-high (year of education: *M* = 15.1, *SD* = 3.1; degree = 14, high school diploma = 16, junior high school diploma = 2), as well as the socioeconomic status measured according to the Hollingshead’s classification (Hollingshead 1975; SES index of: *M* = 28.4, *SD* = 15.0).

Immediately after the SCL-90_R compilation, the participants were administered, in sequence, the Wechsler Adult Intelligence Scale—Fourth Edition (WAIS-IV; Wechsler 2008) to measure the Intelligence Quotient, and the Relationship Anecdotes Paradigm interview (RAP; Luborsky and Crits-Christoph 1990) to collect autobiographical accounts on which applying the linguistic measures of referential competence. During test administration, there was a break of 10 min in the middle or at the end of the WAIS-IV test. The RAP interview was audiotaped with the participants’ consent so that it could then be analyzed linguistically. The administrators were two licensed psychologists trained in the administration of the WAIS-IV and the RAP interview.

### 2.2. Instruments

- *Symptom Check List Revised* (SCL-90-R; Derogatis 1992). SCL-90_R is a widely used tool to screen psychological symptoms measured on nine dimensions: somatization, obsessivity-compulsivity, interpersonal sensitivity, depression, anxiety, hostility, phobic anxiety, paranoid ideation, and psychoticism. Based on the scores in these dimensions, three global indices can be calculated: Global Severity Index (GSI), Positive Symptom Total (PST), and Positive Symptom Distress Index (PSDI). SCL-90-R is a self-report questionnaire consisting of 90 items that refer to symptoms of psychological distress experienced in the last week. We used the Italian version of SCL-90-R that has showed good reliability and validity (Preti et al. 2019).
- *Wechsler Adult Intelligence Scale*—*Fourth Edition* (WAIS-IV; Wechsler 2008). WAIS-IV is the most widely used test for comprehensive assessment of the cognitive abilities of adolescents and adults between the ages of 16 and 90. It is composed by a general intellectual ability scale, the Full Scale of Intelligence Quotient (FSIQ), and by four composite scores: the Verbal Comprehension Index (VCI), including three core subtests (Similarities, Vocabulary, Information) and one supplemental (Comprehension); the Perceptual Reasoning Index (PRI), including three core subtests (Block Design, Matrix Reasoning, Visual Puzzles) and two supplemental (Picture Completion, Figure Weights); the Working Memory Index (WMI), including two core subtests (Digit Span, Arithmetic) and one supplemental (Letter–Number Sequencing); and the Processing Speed Index (PSI), including two core subtests (Symbol Search, Coding) and one supplemental (Cancellation). The administration manual indicates to administer the ten core subtests with the option to replace them with the supplemental subtests, if necessary to compensate for invalid subtest administration or for specific reasons guiding the assessment. In our study, we replaced for VCI the Information subtest with the Comprehension subtest, for WMI the Arithmetic subtest with Letter–Number Sequencing subtest, and for PRI, we added the Picture Completion. These substitutions and addition were made to administer the subtests that most closely connected sensations from multiple sensory domains, in accord with the focus of the study. The WAIS-IV test has been extensively tested in terms of validity and reliability in various cultural contexts, including Italy (Orsini and Pezzuti 2013, 2015; Wechsler 2008).
- *Relationship Anecdotes Paradigm interview* (RAP; Luborsky and Crits-Christoph 1990). The RAP interview was developed to collect, even outside of psychotherapy sessions, accounts of relational episodes from which to infer people’s central transferential themes, applying the Core Conflictual Relationship Theme coding method. Interviewees are asked to recount ten significant episodes of their lives that involve an interaction between themselves and others. For each episode, they are invited to specify when it occurred, with whom it occurred, something about what the other person said or did, and what happened in the end. For the purposes of the present study, RAP was administered to collect from participants meaningful autobiographical narratives on which to apply the linguistic measures related to variables under investigation. Evidence for validity of the RAP potential to evoke significant personal accounts was provided by Barber et al. (1995).

### 2.3. Linguistic Measures

Autobiographical narratives collected through the RAP Interview were analyzed linguistically by the *Discourse Attributes Analysis Program* (DAAP; Maskit and Murphy 2011; Maskit et al. 2012; Maskit 2021). DAAP is designed to compare any type of text to word lists or dictionaries. They can be unweighted, and the output of their application is the proportion of words covered by dictionaries in the texts; other dictionaries can be weighted, i.e., the listed words that relate more strongly to a specific construct will have greater “weights” than the words that are less strongly related to it. In this case, the output is the average weight of the words present in the texts i.e., the degree of intensity of the presence of a certain construct in the texts.

Within the multiple code theory perspective, Bucci and colleagues (Bucci 2021a; Bucci and Maskit 2006; Bucci et al. 2004; Maskit 2021; Zhou et al. 2021) have developed and validated several dictionaries that are computerized linguistic measures of different aspects of the referential process, detectable trough the language characteristics of conversations, whether they arise in psychotherapy sessions or in other interpersonal contexts. For the purposes of this study, we applied five dictionaries among those developed and validated for the Italian language (Bonfanti et al. 2008; Di Trani et al. 2018; Mariani et al. 2013; Negri and Ongis 2021; Negri et al. 2018, 2020):

- *Italian Weighted Referential Activity Dictionary* (IWRAD). IWRAD contains a list of 9596 frequently used Italian words, each assigned a weight between 0 (low) and 1 (high), with 0.5 as the neutral value. A high score represents a high level of referential activity, which corresponds to a high level of concreteness, specificity, clarity, and imagery in the speech sample. The referential activity can be defined as the degree to which the speaker or writer is able to translate their emotional, visceral, and relational experience into words, so as to evoke corresponding experiences in the listener or in the reader (Bucci 2000; Bucci et al. 2004). It is a measure of emotional involvement and the connection between words and the emotional experience. Most of the IWRAD score depends on stylistic rather than content aspects, that is, on how words (and especially function words) are used rather than their content.
- *Italian Weighted Reflection and Reorganization List (IWRRL).* The IWRRL is a measure of the reflection and reorganizing language function that can be defined as the degree to which the speaker is trying to recognize and understand the emotional significance of an event or set of events in their own or someone else’s life, or in a dream or fantasy; it is not about abstract reflection, but rather a person’s reasoning related to an event that has been vividly experienced (Bucci 2021b; Maskit 2021; Negri et al. 2018; Zhou et al. 2021). IWRRL is an index of personal elaboration of emotional experiences and contains a list of 1633 frequently used Italian words, each assigned a weight between 0 (low) and 1 (high), with 0.5 as the neutral value. In the IWRRL, most of the score depends on stylistic rather than content aspects, that is, on how words are used rather than their content.
- *Italian Sensory Somatic Dictionary* (ISenSD). ISenSD is a list of 1926 Italian words related to the body and bodily activities, and to sensory processes or symptoms. The number of ISensD words in a speech sample is a measure of the arousal of bodily and emotional sensations and feelings. It is an unweighted dictionary, and the output is the proportion (ranging from 0 to 1) of words covered by the dictionary in the texts examined.
- *Italian Reflection Dictionary (IRefD).* IRefD is a measure of the abstract reflection present in speech. It consists of a list of 908 Italian words that refer to cognitive or logical functions, and to communication processes that imply the use of cognitive functions; if it is not associated with a narrative with high IWRAD, the IRefD is often indicative of an intellectualizing and defensive style of the speaker (Bucci 2021b; Mariani et al. 2013; Maskit 2021). IRefD is an unweighted dictionary, and the output is the proportion (ranging from 0 to 1) of words covered by the dictionary in the texts analyzed.
- *Italian Affect Dictionary (IAffD).* The IAffD consists of 1786 Italian words. It is a measure of the degree to which the speaker uses words to name and label feelings and emotions; it is a measure of emotional presence in the discourse, but also a defensive and distancing action toward the emotional engagement because the speaker uses abstract words to name affects, rather than describing the emotional experience in a vivid, specific, and concrete way (Bucci 2021b; Bucci et al. 2016; Mariani et al. 2013; Maskit 2021; Negri and Ongis 2021). IAffD are further subclassified as Italian Positive Affect Dictionary (IPAffD), Italian Negative Affect Dictionary (INAffD), and Italian Neutral Affect Dictionary (IZAffD). IAffD and its sub-dictionaries are unweighted dictionaries, and their output are the proportion (ranging from 0 to 1) of words covered by the dictionary in the texts analyzed.

### 2.4. Data Analyses

As preliminary analyses, we tested for possible effects of sociodemographic variables (gender, age, education, socio-economicus status) on the dependent and independent variables (WAIS Indices, IWRAD, IWRRL, ISenSD, IRefD, IAffD): we applied Student’s t-test on the scores of male and female participants, and we calculated Pearson’s correlation coefficients between age, SES, years of education and the variables under analysis.

To test the hypothesis, we first calculated Pearson’s correlation coefficients between linguistic measures of autobiographical narratives and the scores obtained on WAIS-IV scales; in this analysis, we entered as controls the sociodemographic variables found to be associated with dependent and independent variables. Second, based on the correlations found to be significant, a linear regression analysis was performed using the linguistic dimensions measured on the autobiographical narratives as predictors of the WAIS-IV scores. All analyses were conducted using *Jamovi* software (2021).

## 3. Results

The participants obtained scores close to the average on the WAIS-IV (*M* = 102), with an important variability (*SD* = 13.1) as the minimum score obtained in the FSIQ was at the lower limit of the average (FSIQ = 70) and the maximum score reached was well above the average (PRI = 141) (see Table 1). Between males and females, there were no significant differences in any of the overall or composite scores, as well as in the individual subtests. Age, years of education, and socio-economic status did not correlate with WAIS-IV scores, except for the WMI and its Letter–Number Sequencing subtest, which correlated negatively with age, respectively, *r* = −.508, *p* = .006, and *r* = −.666, *p* < .001.

In the autobiographical narratives, participants showed levels of referential activity (IWRAD) close to the average (*M* = .5050, *SD* = .0027) as well as for reflecting and reorganizing style scores (*M* = .5440, *SD* = .0027). The application of the sensory and somatic (ISensD), refection (IRefD), and affect (IAffD) dictionaries yielded average proportions respectively of .0404, .0300, .0343, which means that, on average, 4.0%, 3.0%, and 3.4% of the words in the autobiographical stories belong to these dictionaries (see Table 1). No significant correlations were found between these linguistic measures, except between the two measures related to different aspects of reflection, namely, abstract reflection as measured by words referring to logical or cognitive functions (IRefD) and reflective and reorganizational language style (IWRRL), *r* = −.371, *p* = .036. Male participants told narrative with significantly higher levels of referential activity of narratives (*t*_(30)_ = 2.567, *p* = .015, *d* = 0.955; IWRAD: Male = .5064, Female = .5036) while no difference between males and females was found in the usage of all other dictionaries. Correlations between linguistic measures and age, years of education and socio-economic status were not found, except for IWRAD and year of education, *r* = −.516, *p* < .004.

Concerning the hypothesis of the study the WAIS-IV scores did not correlate with referential activity (IWRAD), reflection and reorganization function (IWRRL), abstract reflection (IRefD), and affect-related words (IAffD and relative sub-dictionaries), while the sensory somatic dictionary (ISenSD) positively correlated with the overall scores of intelligence (FSIQ: *r* = .558, *p* = .002), the working memory scores (WMI: *r* = .531, *p* = .003), and the subtests of Digit Span (*r* = .487, *p* = .007) and Letter–Number Sequencing (*r* = .429, *p* = .020). The computation of correlation coefficients was controlled for years of education and age.

**Figure 1:**
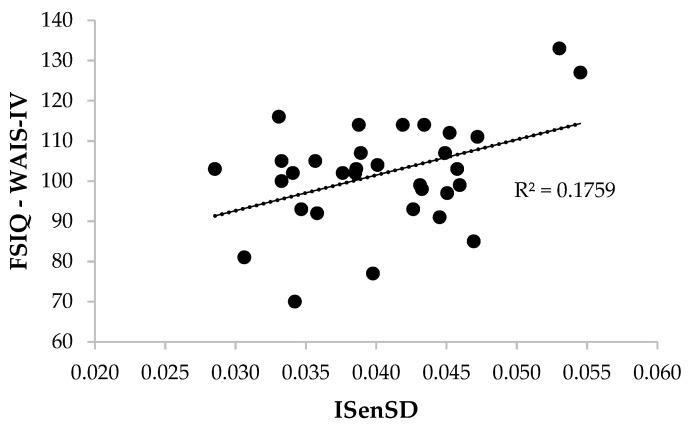
Scatter plot of ISenSD and FSIQ (WAIS-IV) scores.

**Figure 2:**
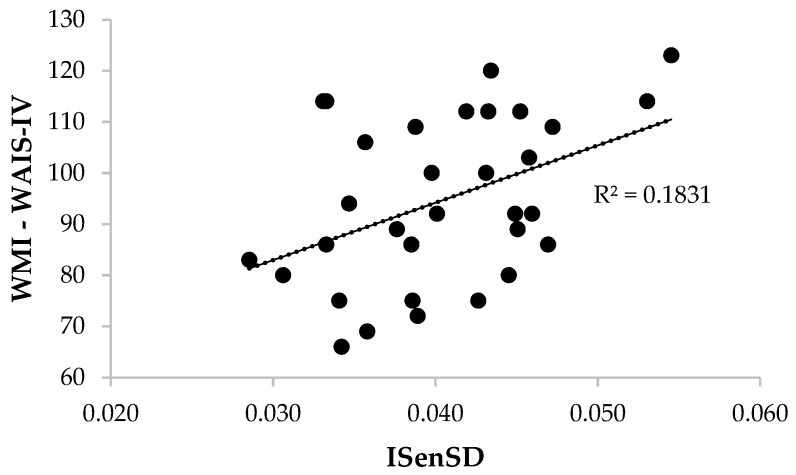
Scatter plot of ISenSD and WMI (WAIS-IV) scores.

Two linear regression analyses indicated that the participants’ usage of sensory somatic words (ISenSD) in their autobiographical narratives explained 17.6% of the variance of the FSIQ scores (R^2^ = .176, *F*_(1,30)_ = 6.40, *p* = .017) and 18.3% of the variance of the WMI scores (R^2^ = .183, *F*_(1,30)_ = 6.73, *p* = .015) (see Figure 1 and Figure 2). Specifically, the proportion of sensory and somatic words (ISenSD) predicted the overall levels of intelligence (FSIQ), β = 0.419, 95% CI [0.081–0.758], *t* = 2.53, *p* = .017, *d* = 1.062, and the working memory scores (WMI), β = 0.428, 95% CI [0.091–0.765], *t* = 2.59, *p* = .015, *d* = 1.089.

In sum, the hypothesis that language referring to bodily, sensory, and somatic functions (SenSD) is to some extent an indicator of intellectual abilities (IQ and WMI) as intrinsically embodied, is supported by the results. Instead, referential activity, i.e., the level of interconnection between the subsymbolic (perceptual and nonverbal) and symbolic (verbal) processing systems, which is revelated more by linguistic style than by the type of words used, was not associated with the level of intelligence. Similarly, the other indices of referential competence related to more abstract domains of experience (affect, abstract reflection, and reorganizing and reflecting linguistic style) did not correlated with intelligence measures.

## 4. Discussion

The measures of referential process that have been used concern various referential domains. Only the measure of the sensory and somatic sensations dictionary was found to correlate with intelligence quotient. The participants who used more words referring to bodily experiences and sensory processes while describing their own relations and emotions showed higher intelligence scores. On the contrary, words related to other more abstract domains (abstract reflection and abstract labels of emotional sensations) or the stylistic use of words in a reflecting way were not associated to participant’s intelligence. We believe that these results support the embodied cognition hypothesis (Barsalou 1999; Clark 1998, 2008; Gallese 2008; Lakoff and Johnson 1980; Nöe 2004; Thompson 2007). The sensorimotor sensations we experience during the movement of our body in the space are the basic elements of sense-making processes, including concepts and language. Several studies and theories (Gallese 2008; Glenberg and Gallese 2012; Gallese and Lakoff 2005; Glenberg 2007; Hauk et al. 2004; Lakoff and Johnson 1980; Tettamanti et al. 2005) have highlighted how the language structure reveals motor concepts and similarities as foundations of every language. For example, Pinker (2007) maintained that almost every abstract word used is based on a practical metaphor, which are also derived from the perception of elements, such as the movement. He defines the human intelligence as a process of metaphoric abstraction that deprives the conceptual structures from their content, and that applies them to new abstract domains. In this sense, having words that can refer to one’s somatic and sensory states and using them to make sense of what is happening in our sensory space helps us to think better and implement more efficient and adaptive behavior, in other words, more intelligent.

Based on this evidence, we can hypothesize that sensorimotor vocabulary can thus be considered both an indirect measure of intelligence and an index of the degree of embodiment of higher cognitive processes, i.e., their connection with sensory and motor systems. If this is true, the dictionary of words related to sensory and somatic sensations (ISenSD; see Table 2 for examples of Italian words belonging to this dictionary; for the English version of the dictionary, see Bucci 2021b, 2021c; Maskit 2021) may represent a tool to measure embodiment and partially fill the lack of tools available to measure this construct in the field of psychological and neuropsychological research. This dictionary can also be a tool to be tested in the educational field for screening children with difficulties in sensorimotor integration and cognitive development.

Another result worth discussing is that the weighted dictionary of referential activity (IWRAD) did not correlate with the IQ scores. The referential activity is operationalized as the degree to which the speaker or writer is able to translate their emotional, visceral, and relational experience into words, so as to evoke corresponding experiences in the listener or in the reader (Bucci 2000; Bucci et al. 2004). Thus, the more specific, concrete, detailed, and vivid the speech is, the more it has high referential activity. In other words, the referential activity is the extent of connection between words and nonverbal experience. According to the embodied cognition hypothesis, we should expect this measure to correlate with intelligence but, as in Bucci and Freedman’s (1978) study from which we have drawn inspiration, intelligence and referential activity did not correlate. We agree with the authors that this happens because WAIS-IV remains focused on measuring cognitive ability in abstract, decontextualized terms and based on verbal ability; it is therefore more a measure of rational reasoning and logical connection between words. On the other hand, instead, the referential activity is a measure of the referential competence, i.e., of the ability to connects words to objects, events, relationships, etc. Moreover, most of the IWRAD score depends on stylistic rather than content aspects, that is, on how words (and especially function words) are used to evoke a situated and concrete experience rather than their content that tend to represent a simplification and abstraction of the experience (see Table 2 for examples of Italian words belonging to this dictionary; for the English version of the dictionary, see Bucci 2021b, 2021c; Maskit 2021). For this reason, it would be interesting to investigate the relationship between referential activity and intelligence using more situated, specific, and nonverbal measures of the cognitive abilities.

Finally, it is intriguing that among the WIAS-IV indices, the only one that, in addition to IQ, correlated with sensory and somatic dictionary was working memory (WMI). It is a finding that suggests that working memory processes are strictly related to bodily processes such as keeping pace and repeating action in time and space (e.g., Kang et al. 2020; Smyth et al. 1998).

The results of the present study, however, should not be considered conclusive. They certainly need to be corroborated by other evidence by applying, for example, the research design to larger samples and to populations with differentiated levels of intelligence. The dictionaries of the referential competence should also be applied in conjunction with different measures of intelligence and embodiment to produce more robust conclusions. Despite the exploratory and pilot nature of the study, we think that the results presented here open a novel way to the study of embodied cognition that can be promising because the collection of people’s linguistic productions is relatively simple to implement and analyze. The results of the present study on the association between intelligence and somatic and sensory vocabulary are a proof of this viability and add to those already numerous in support of the hypothesis that sensorimotor schemas have an intrinsic role in language and cognition.

## Figures and Tables

**Table 1:** Descriptive statistics of WAIS-IV scores and language measures.

			Males (n = 11)	Females (n = 21)	Total (N = 32)
WAIS-IV	FSIQ	M *(SD)*	102.0 (*10.7)*	102.0 *(14.4)*	102.0 (*13.1)*
		Min–Max	77–114	70–133	70–133
	VCI	M *(SD)*	102.0 (*10.6)*	105.0 (*17.5)*	104.0 (*15.3)*
		Min–Max	78–118	65–127	65–127
	PRI	M *(SD)*	107.0 *(16.0)*	103.0 *(16.3)*	105.0 *(16.1)*
		Min–Max	67–127	75–141	67–141
	WMI	M *(SD)*	99.5 *(15.0)*	92.1 *(16.6)*	94.7 *(16.2)*
		Min–Max	75–120	66–123	66–123
	PSI	M *(SD)*	96.1 *(16.4)*	102.0 *(8.72)*	99.7 *(12.0)*
		Min–Max	67–128	89–119	67–128
Linguistic Measures	IWRAD	M *(SD)*	.5060 *(.0029)*	.5040 *(.0023)*	.5050 *(.0027)*
		Min–Max	.5020–.5110	.5000–.5090	.5000–.5110
	IWRRL	M *(SD)*	.5450 *(.0019)*	.5440 *(.0021)*	.5440 *(.0021)*
		Min–Max	.5410–.5470	.539–.5470	.5390–.5470
	ISenSD	M *(SD)*	.0404 *(.0059)*	.0404 *(.0065)*	.0404 *(.0062)*
		Min–Max	.0285–.0472	.0306–.0545	.0285–.0545
	IRefD	M *(SD)*	.0328 *(.0105)*	.0286 *(.0070)*	.0300 *(.0084)*
		Min–Max	.0224–.0602	.0164–.0456	.0164–.0602
	IAffD	M *(SD)*	.0344 *(.0059)*	.0342 *(.0069)*	.0343 *(.0065)*
		Min–Max	.0257–.0437	.0254–.0457	.0254–.0457

**Table 2:** Examples of words belonging to ISenSD and IWRAD.

Dictionary		Examples of Words
**ISenSD**	Actions	*mordere, allattare, respirare, piangere, ballare, scappare, fare esercizio, cadere, combattere, gesticolare, alzarsi, maneggiare, abbracciare, saltare, zoppicare, correre, urlare, gridare, sciare, fumare, sculacciare, sputare, sudare, girare, camminare* biting, breastfeeding, breathing, crying, dancing, escaping, exercising, falling, fighting, gesticulating, getting up, handling, hugging, jumping, limping, running, screaming, skiing, smoking, spanking, spitting, sweating, turning, walking
	Sensory sensations	*scalzo, colazione, cena, bere, mangiare, energico, grasso, sapore, fame, ipersensibile, pranzo, piacevole, postura, pugno, rilassato, sensazione, sensi, sessuale, tremare, sonno, odore, liscio, morbido, stimolato, puzza, gusto, solletico, toccare, viscerale* barefoot, breakfast, dinner, drinking, eating, energic, fat, flavor, hunger, hypersensitive, lunch, pleasant, posture, punch, relaxed, sensation, senses, sexual, shaking, sleep, smell, smooth, stimulated, stink, taste, tickle, touching, visceral
	Negative sensations	*ansioso, freddo, colica, collasso, contrazione, distrutto, svenimento, febbre, influenza, malattia, nausea, nervoso, dolori, pallido, panico, paralizzato, faringite, sciatica, scoliosi, sofferenza, sintomo, tachicardia, lacrime, brivido, stanco, scomodo, vomito* anxious, cold, colic, collapse, contraction, destroyed, fainting, fever, flu, illness, nausea, nervous, pains, pale, panic, paralyzed, pharyngitis, sciatica, scoliosis, suffering, symptom, tachycardia, tears, thrill, tired, uncomfortable, vomiting
	Bodily parts	*braccio, culo, schiena, palle, pancia, sangue, corpo, seno, petto, colon, cornea, feci, occhio, viso, carne, genitali, capelli, mani, testa, cuore, rene, lineamenti, fegato, lombare, polmone, mestruale, unghie, ovaie, pipì, fisico, retto, scheletro, pelle, stomaco* arm, ass, back, balls, belly, blood, body, breast, chest, colon, cornea, excrements, eye, face, flesh, genital, hair, hands, head, heart, kidney, lineaments, liver, lumbar, lung, menstrual, nails, ovaries, pee, physique, rectal, skeleton, skin, stomach
**IWRAD**	High weight	*accidentale, appetito, baciare, bava, coltelli, impietrito, maledetto, martello, mortificata, odore, respirazione, sottili, stupore, sudo, urla, verme, vomito* accidental, appetite, kissing, slime, knives, petrified, dammed, hammer, mortified, smell, breathing, subtle, astonishment, sweat, screams, worm, vomit
	Medium weight	*un, tutti, già, comunque, sono, corpo, però, può, per, da, hanno, io, nella, è, lei, mio, devo, niente, altri, probabilmente, cioè, il, dunque, questo, molto, con, sì, tu, capisci* a, all, already, anyway, are, body, but, can, for, from, have, I, in, is, she, my, must, nothing, others, probably, that is, the, then, this, very, with, yes, you, you know
	Low weight	*banalità, dica, dimostra, dipenderà, entità, lealtà, lineare, mediocre, nuvola, propriamente, quotidianità, scontata, sfaccettature, simili, superficiali, poiché, eppure, senza* banality, says, demonstrates, depend on, entity, loyalty, linear, mediocre, cloud, properly, everyday life, obvious, facets, similar, superficial, since, yet, without

## Data Availability

The data presented in this study are available on request from the corresponding author.
